# Characterization of the differences in the cyclopiazonic acid binding mode to mammalian and *P. Falciparum* Ca^2+^ pumps: A computational study

**DOI:** 10.1002/prot.24734

**Published:** 2015-01-10

**Authors:** Daniele Di Marino, Ilda D'Annessa, Andrea Coletta, Allegra Via, Anna Tramontano

**Affiliations:** 1 Department of Physics, Sapienza UniversityP.Le Aldo Moro 5, Rome, 00185, Italy; 2 Department of Biology, University of “Tor Vergata,”via Della Ricerca Scientifica Snc, Rome, 00133, Italy; 3 Istituto Pasteur-Fondazione, Cenci Bolognetti, Sapienza UniversityP.Le Aldo Moro 5, Rome, 00185, Italy

**Keywords:** SERCA, pfATP6, malaria, CPA, homology modeling, molecular dynamics, MM-GBSA

## Abstract

Despite the investments in malaria research, an effective vaccine has not yet been developed and the causative parasites are becoming increasingly resistant to most of the available drugs. PfATP6, the sarco/endoplasmic reticulum Ca2+ pump (SERCA) of *P. falciparum*, has been recently genetically validated as a potential antimalarial target and cyclopiazonic acid (CPA) has been found to be a potent inhibitor of SERCAs in several organisms, including *P. falciparum*. In position 263, PfATP6 displays a leucine residue, whilst the corresponding position in the mammalian SERCA is occupied by a glutamic acid. The PfATP6 L263E mutation has been studied in relation to the artemisinin inhibitory effect on *P. falciparum* and recent studies have provided evidence that the parasite with this mutation is more susceptible to CPA. Here, we characterized, for the first time, the interaction of CPA with PfATP6 and its mammalian counterpart to understand similarities and differences in the mode of binding of the inhibitor to the two Ca2+ pumps. We found that, even though CPA does not directly interact with the residue in position 263, the presence of a hydrophobic residue in this position in PfATP6 rather than a negatively charged one, as in the mammalian SERCA, entails a conformational arrangement of the binding pocket which, in turn, determines a relaxation of CPA leading to a different binding mode of the compound. Our findings highlight differences between the plasmodial and human SERCA CPA-binding pockets that may be exploited to design CPA derivatives more selective toward PfATP6. Proteins 2015; 83:564–574. © 2015 The Authors. Proteins: Structure, Function, and Bioinformatics Published by Wiley Periodicals, Inc.

## INTRODUCTION

Malaria puts about half the world's population at risk of infection and causes >800,000 deaths each year. Five species of a protozoan parasite of the *Plasmodium* genus cause malaria. *P*. *falciparum*, the most deadly species, has become resistant to some of the most commonly used drugs, such as chloroquine, antifolates, and aminoalcohols, and there is preliminary evidence that resistance to artemisinins may also be emerging.^1^

Ca^2+^-ATPases are calcium pumps found in bacteria, archaea and eukaryotes that transfer Ca^2+^ across membranes into intracellular stores at the expense of ATP hydrolysis.^2^ Because of their ability to trigger apoptosis, Ca^2+^-ATPases from different species are potential therapeutic targets for the treatment of cancer and parasitic infections.^3^ PfATP6 shows a high degree of sequence identity with proteins of the mammalian sarco/endoplasmic-type Ca^2+^-ATPase (SERCA) family^4^ (40% global sequence identity with the rabbit SERCA1a). SERCA1a is a membrane protein residing in the sarco- and endoplasmic reticulum (SR/ER). It is composed of three cytoplasmic domains and 10 transmembrane (TM) helices (M1-M10). The three cytoplasmic domains, referred to as nucleotide binding (N), phosphorylation (P), and actuator (A) domains, undergo large motions on ion transport.^5^ The A-domain contains the TGES sequence motif, which plays an important role in the catalytic cycle, whereas the P-Domain contains Asp351, the phosphorylation of which induces a large conformational change that facilitates the Ca^2+^ transport.^6^ Both the A- and P-domains are connected to the TM region through a linker region.^6^ The A-domain, which is connected to the M1, M2, and M3 trans-membrane helices through three flexible regions, is the actuator of the trans-membrane gating mechanism, which regulates Ca^2+^ binding and release.^6^ Active calcium transport is achieved by alternating four states: The Ca^2+^-E1-P* state,^6^ where the two binding sites have high affinity for Ca^2+^ and face the cytoplasmic side of the SR/ER membrane; The E2P open state, where the binding sites have low affinity for Ca^2+^, high affinity for protons,^6^ and face the SR/ER lumen; The closed E2-P*, which is proton occluded; finally, the E2-ATP state, where a modulator ATP is bound.^6^ The three-dimensional (3D) structure of SERCA1a has been determined in all four states of the reaction cycle.

Several compounds have already been shown to bind and inhibit SERCA1a by locking its structure in a defined conformational state.^7^ For example, Thapsigargin (TG), cyclopiazonic acid (CPA), and 2,5-di-(tert-butyl) hydroquinone (BHQ) block the protein in an E2-like state, whereas 1,3-dibromo- 2,4,6-tri (methylisothiouronium) benzene stabilizes an E1-P*-like conformation.^7^

PfATP6 contains the motifs important for calcium transport, such as the nucleotide-binding site, two high-affinity Ca^2+^ binding sites, and the phosphorylation site. Moreover, it has been observed that the ER-like Ca^2+^ storage of *P. falciparum* trophozoites is inhibited by CPA^8^ and that PfATP6 is sensitive to this compound.^9^ CPA has been isolated from stored grain and cereal products infected with the fungus *Penicillium* cyclopium.^10^ The SERCA inhibition mechanism by CPA and its cytotoxicity have been extensively studied as a consequence of food poisoning following the ingestion of contaminated feed.

CPA acts by occluding the SERCA1a calcium channel and its binding site is different from that of TG and partially overlaps with that of BHQ.^11^

Although the sequence identity is very high in the trans-membrane region between mammalian SERCA1a and PfATP6, there is a non-conservative amino acid mutation (Glu to Leu) at SERCA1a position 255 (_m_255) located on helix M3, corresponding to position 263 (_p_263) in PfATP6. The role of this mutation has been studied in relation to the inhibitory effect exerted by artemisinin^12^ and, recently, Pulcini and colleagues have shown that *P. falciparum* with the PfATP6 Leu263Glu mutation is more susceptible to CPA, even though the amino acid in position 263 is not involved in the direct binding of the compound, as it can observed in the rabbit SERCA1-CPA structural complex (PDB: 3FGO).

Noticeably, despite the differences between SERCA1a and pfATP6, the CPA binding affinity is in the micromolar range for both proteins.^4^,^13^–^15^ More specifically, the IC_50_ values of CPA inhibition range between 0.6 and 10 μ*M* for SERCA1a and between 0.4 and 3 μ*M* for pfATP6, depending on the experimental conditions.

Because of its toxicity, CPA itself is not indicated as an antimalarial drug but, thanks to its inhibitory potency against the target, it is a promising lead compound for the design and development of derivatives more specific toward PfATP6.

In this work, the molecular mechanism of CPA binding to wild type SERCA1a and PfATP6 was investigated, allowing us to understand how the amino acid at position _m_255 (_p_263) affects the binding of CPA. In particular, we performed two Molecular Dynamics (MD) simulations of 185 ns each followed by MM-GBSA calculations for both CPA-PfATP6 and CPA-SERAC1a complexes, aimed at studying their interaction energy.

The MM-GBSA approach made it possible to quantify the energy contribution of each amino acid composing the binding site and to determine the structural differences of CPA binding between SERCA1a and PfATP6. Our findings offer a starting point for the design of CPA derivatives specific for PfATP6.

## MATERIAL AND METHODS

### Homology modeling

The three-dimensional (3D) structure of PfATP6 was modeled using the following procedure: (1) to identify evolutionary conserved regions, a multiple alignment of the PfATP6 sequence (UniProt AC Q08853) and its homologous was produced using T-Coffee^16^ and manually inspected (Supporting Information Fig. S1). (2) The template search was performed using the HHsearch method for HMM–HMM comparison employed by the HHpred server,^17^ which identified, as templates, the X-ray structures of the rabbit SERCA1a protein in several conformations. Among these, we selected as best template a SERCA1a structure co-crystallized with a molecule of CPA and an Mn^2+^ heavy atom in the EP2-like conformation (PDB: 3FGO^5^). (3) The target and template sequences were re-aligned (40% sequence identity) with the Stretcher program (http://cbsuapps.tc.cornell.edu/stretcher.aspx) and further adjusted manually (Supporting Information Fig. S2). The target-template alignment highlighted two long insertions (LCRs) in the cytoplasmic region of PfATP6 (Supporting Information Fig. S2). (4) The template structure and the PfATP6 sequence depleted of the LCRs were used as input to the standalone version of Swiss-PdbViewer v4.1^18^ to build a single model of the transmembrane and cytoplasmic conserved regions of the plasmodial protein. The model construction was based on the target-template alignment previously obtained. (5) The structure of the LCRs was predicted *ab initio* using the I-TASSER web server.^19^ I-TASSER generated five models with very similar C-score values. Among these, we selected the LCR conformation best satisfying the geometrical restraints of the stem regions (the main chain atoms that precede and follow the loop) in the PfATP6 homology model. (6) The predicted insertions were added to the PfATP6 model using Swiss-PdbViewer v4.1.^18^ (7) The Swiss-PdbViewer's rotamer library was used to change amino acids side-chains and the Swiss-PdbViewer's loops library to choose the best fitting loops. (8) To validate the model, we used QMEANBrane, a local model quality estimation method for membrane proteins that combines statistical potentials trained on membrane protein structures with a per-residue weighting scheme.^20^ (9) To regularize the structure, that is, avoid clashes and wrong side-chains positioning, a round of 5000-step minimization was performed on the PfATP6 modeled structure in vacuum using the Steepest Descent algorithm.^21^

The final model has been deposited into the PMDB Protein Model Database^22^ and can be retrieved using the PM0079760 ID.

### Protein insertion into the membrane and MD simulation pre-processing

The final 3D model of PfATP6 and the crystal structure of the rabbit SERCA1a (PDB: 3FGO), which include three crystallographic water molecules coordinating the Mn^2+^ ion, were inserted in a pre-equilibrated lipid bilayer of 409 1-palmitoyl-2-oleoyl-sn-glycero-3-phosphocholine (POPC) molecules using the CHARMM-GUI web server.^23^ The PfATP6 system was solvated and neutralized with 62.304 TIP3P water molecules and 7 Na^+^ counter-ions, whereas the SERCA1a system with 58.245 water molecules and 15 Na^+^ counter-ions.

The PfATP6 modeled structure and the SERCA1a-CPA X-ray complex (PDB: 3FGO) were superimposed on the Cα atoms of the conserved regions between the two proteins. Subsequently, CPA was transferred from the X-ray structure into the PfATP6 model. A first round of 10,000-step minimization was performed on both PfATP6 and SERCA1a systems using the Steepest Descent algorithm.^21^

The topology of the protein moiety of both the CPA-SERCA1a and CPA-PfATP6 systems was generated with the AMBER tleap program using the ff10 force field.^24^ CPA and lipid topologies were determined as described in the Molecular Mechanics section. The Mn^2+^ ion force field parameters were retrieved from the AMBER parameter database (http://www.pharmacy.manchester.ac.uk/bryce/amber).

The two systems were enclosed in a periodic box with the lipid bilayer oriented in the xy-plane, and subsequently equilibrated in two steps. In the first step the protein + CPA + Mn^2+^ + the crystallographic water molecules were restrained using Cartesian harmonic constraints with a constant of 1 Kcal mole^−1^ Å ^−2^, whereas the rest of the system was equilibrated in the NPT ensemble using six steps of 100 ps (time-step = 1 fs) and a temperature ramp ranging from 50 to 300 K. In the second step, the constraints on the systems were relaxed by decreasing the harmonic constant from 1000 to 0 in four steps.

### Molecular dynamics simulation

After verifying the stability of the box dimensions, the production run was performed in the NVT ensemble, using the CUDA-enabled version of Amber12 PMEMD.^24^ A cut-off of 10 Å was used for van der Waals and electrostatic interactions; long-range electrostatic interactions were taken into account with the PME algorithm using the default options of PMEMD.^24^ The final trajectory was converted to the .xtc format and standard MD analyses were performed with the Gromacs 4.5 tools.^25^ Visual analyses of the final modeled structures were carried out with Visual Molecular Dynamics.^26^ Plots were obtained with the Grace-5.1 program (http://plasma-gate.weizmann.ac.il/Grace/) and all images generated using PyMol^27^ (http://www.pymol.org) and UCSF Chimera.^28^

The dynamic cross-correlation (DCC) maps of the systems were determined with in-house software, only taking into account the Cα atom coordinates of the proteins since they contain enough information to describe the largest system motions. The elements of the DCC map (*C*_ij_) were computed as:


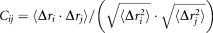
(1)

where Δ*r_i_* is the displacement from the mean position of the *i*th atom and the 〈 〉 indicates the time average over the whole trajectory. *C_ij_* values range between −1 and +1. Positive *C_ij_* values indicate a correlated motion between residues *i* and *j* (i.e., the residues move in the same direction). Negative values of *C_ij_* indicate an anti-correlated motion between residues *i* and *j* (i.e., they move in opposite directions).

### Molecular mechanics

**Figure 1:**
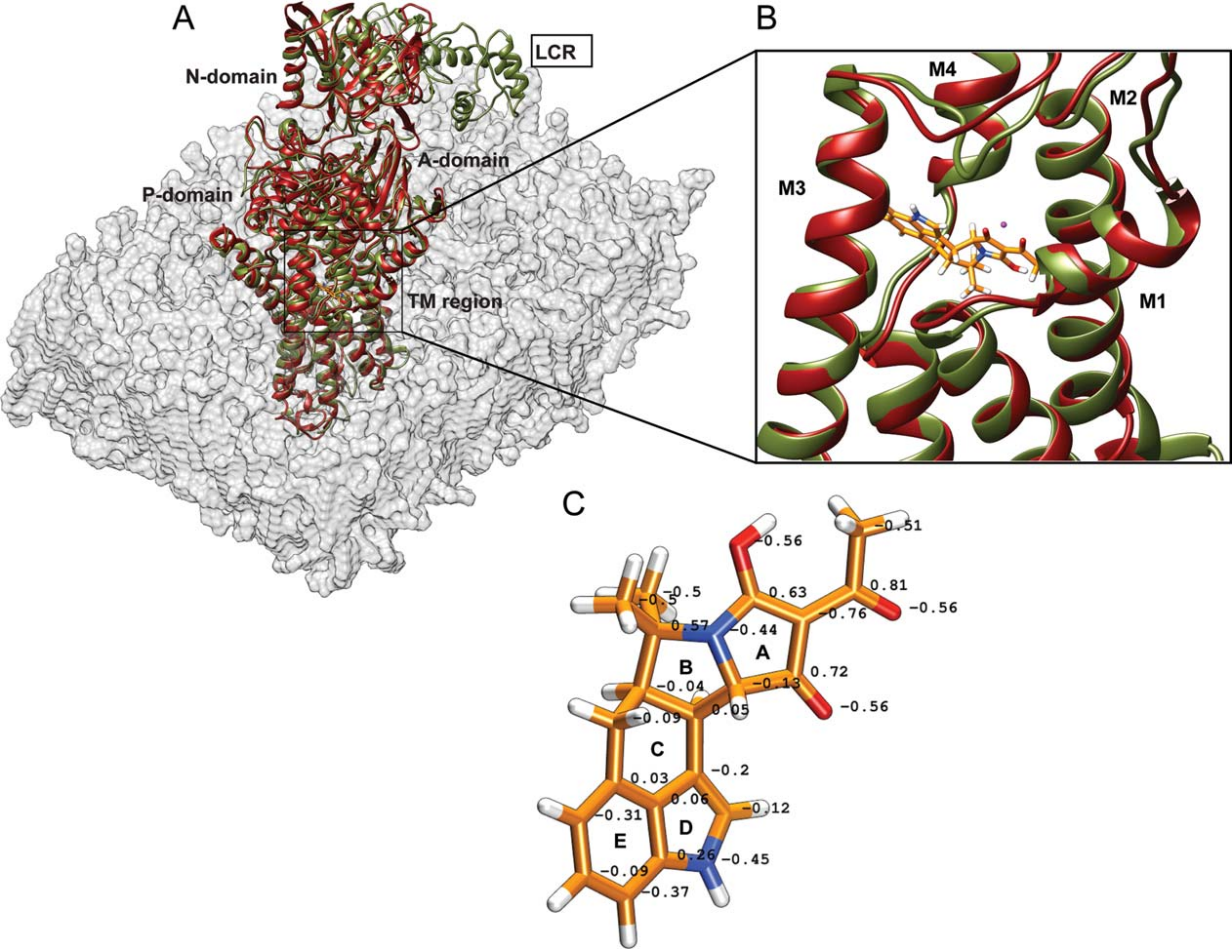
(A) Superposition of the rabbit SERCA1a X-ray structure (in red) and the three-dimensional model of PfATP6 (in green) inserted in the lipid bilayer (in light grey). (B) Superimposition of the four trans-membrane helices M1-M4 hosting the CPA binding site shown in red and in green for SERCA1a and PfATP6, respectively. (C) The structure and charge distribution of CPA. Quantum chemistry calculations were used to determine the atom point charges. Nitrogen atoms are in blue, oxygen atoms in red, hydrogen atoms in white. [Color figure can be viewed in the online issue, which is available at http://wileyonlinelibrary.com.]

The chemical structure of the Cyclopiazonic acid (CPA) [Fig. 1(C)] was pre-processed with Molden 5.0.^29^ The structure was geometrically optimized in vacuum at the HF/6-31G* level of theory. Using these conformations, the atom point charges were fitted with the RESP procedure using a B3 LYP/6-31G* electron density. The AMBER topologies for the compound were prepared using the antechamber tools with the General Amber Force Field (GAFF) included in AmberTools12.^24^ The parameterization of the POPC lipids was obtained using the Glycam-06.h force field.^30^ The parameters thus obtained were used to generate the AMBER topologies for the lipids. The partial charges of the fatty acid moieties were calculated using the RESP method. All quantum chemistry calculations were performed with Gaussian09 (http://www.gaussian.com/g_misc/g03/citation_g03.htm).

### MM-GBSA

MM-GBSA analyses were carried out on the original Amber trajectories after water and Na^+^ ions removal, and using the ante-MMPBSA.py/MMPBSA.py scripts available in the AmberTools12.^24^ The calculations of the binding energies were performed using the last 40 ns of the trajectories including 200 frames (i.e., one structure every 200 ps).

The free energy of binding was determined with the standard MM-GBSA scheme,^31^ using the Hawkins, Cramer, Truhlar pairwise generalized Born model^32^ implemented in Sander^24^ to take the desolvation free-energy into account.

## RESULTS

### System set up and structural analyses of the MD simulations

The 3D model of PfATP6 was obtained by homology modeling using as a template the structure of the rabbit SERCA-CPA complex in the calcium low affinity EP2-like conformation. The quality of the model was overall good and very good in the TM region, as it can be observed from Supporting Information Figure S1(B), where residues are colored from blue (bad model) to red (good model) according to the accuracy of the prediction.

The sequence alignment between PfATP6 and SERCA1a from rabbit (Supporting Information Fig. S2) shows 40 and 55% sequence identity and similarity, respectively. The only non-conserved regions between PfATP6 and SERCA1a are two long PfATP6 cytoplasmic insertions corresponding to low complexity regions (LCRs) of the plasmodium genome.^33^ Plasmodium LCRs are known to encode for long disordered domains mainly composed of lysine and asparagine residues due to the presence of many AT repeats in the genomic sequence.^34^ These types of regions, the function of which is not fully understood,^33^ are found in most of the plasmodium proteins.^34^

The CPA binding site is located in the trans-membrane (TM) domain, which is very well conserved among different species [Supporting Information Fig. S1(A)]. Remarkably, Glu255 (mammalian numbering) is mutated to Leu only in PfATP6 [Supporting Information Fig. S1(A)]. From now on, we will refer to mammalian SERCA and PfATP6 residues using “m” or “p” subscripts, respectively, that is, _m_Glu255 or _p_Leu263. The 10 trans-membrane helices are arranged in a compact bundle with helices 1 to 4 forming the channel through which the Ca^2+^ ions cross the membrane (Supporting Information Fig. S2).

Figure 1(A) shows the superimposition between the PfATP6 model and the X-ray structure of SERCA1a. The TM domain, P-domain, N-domain and the A-domain are well superimposed whereas, as expected, the PfATP6 LCR regions, which are not superimposable with any SERCA1a domain, protrude out the globular moiety of the plasmodial protein [Fig. 1(A)]. The TM domain of both structures was inserted in a lipid bilayer composed of POPC (1-palmitoyl-2-oleoylphosphatidylcholine) to make the simulation as realistic as possible [Fig. 1(A)]. POPC is the most represented lipid in the sarcoplasmic membrane and has already been used in molecular dynamics simulations of the SERCA proteins.^35^ In the X-ray structure of rabbit SERCA1a, CPA is located between helices M1-M2 and M3-M4 [Fig. 1(B)]. The RMSD of the structural superimposition of PfATP6 and SERCA1a on the Cα atoms belonging to the four helices M1-M2 and M3-M4 is 1.2 Å [Fig. 1(B)]. Given the similarity of the CPA binding pocket in the two structures, we hypothesized that the compound assumes a similar orientation in the two proteins.

Figure 1(C) shows the three-dimensional structure of CPA. The molecule is made up of five rings (labeled from A to E) arranged in a “wavy” conformation [Fig. 1(C)]. It contains two nitrogen atoms: N1, which is placed at the junction between rings A and B, and N2, which is located on ring D. The structure can be divided into three portions: the hydrophobic indole group comprising rings E and D, the central region comprising rings C and B, and the acyl-tetramic acid moiety comprising ring A. The computation of the partial charges for each atom of CPA [Fig. 1(C)] showed that N2 is negatively charged [Fig. 1(C)].

**Figure 2:**
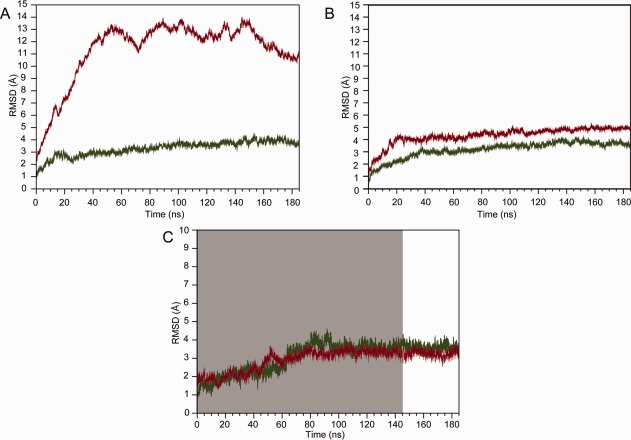
RMSD of the Cα atoms of the SERCA1a (red) and PfATP6 (green) as a function of simulation time. (A) Whole protein and (B) TM regions. (C) RMSD of CPA bound to SERCA1a (red) and PfATP6 (green) as a function of simulation time. The segments of the trajectories not used for the analyses are in grey. [Color figure can be viewed in the online issue, which is available at http://wileyonlinelibrary.com.]

For both the SERCA1a-CPA and PfATP6-CPA complexes, we ran an MD simulation of 185 ns. The RMSD calculated on the protein Cα atoms between the starting and simulated structures is rather different in the two simulations [Fig. 2(A)]: it reaches a maximum value of 14 Å in the simulation of PfATP6, and of ∼3.2 Å in the simulation of SERCA1. Such a high RMSD value reached in the simulation of PfATP6 is substantially due to the presence of the flexible LCRs. These regions are located in the cytoplasmic side of the membrane, far from the CPA binding site, and therefore their high flexibility is unlikely to actually affect the stability of the binding. To verify whether the high flexibility of LCRs affects the motion of the residues belonging to the CPA binding site, we calculated the correlation matrix for all the Cα atoms trajectories in the PfATP6 system (Supporting Information Fig. S3). The correlation matrix showed that there is neither correlation nor anti-correlation between the CPA-binding site residues (highlighted by the black rectangles in Supporting Information Fig. S3) and LCRs residues (red rectangle in Supporting Information Fig. S3).

When the Cα RMSD was only computed for the TM region, we obtained a final RMSD value of ∼4.5 Å and ∼3.2 Å for PfATP6 and SERCA1a, respectively [Fig. 2(B)].

The RMSD of CPA with respect to its starting conformation was also calculated by superimposing the trans-membrane domain [Fig. 2(C)]. In both simulations, CPA deviates with respect to its initial position of a maximum RMSD value of 4 Å [Fig. 2(C)], suggesting that its interaction with the protein is very stable. This result is in line with the experimental data showing that CPA is a strong inhibitor of both PfATP6 and SERCA1a.^9^

In both trajectories, the RMSD reached a very stable plateau after 145 ns [Fig. 2(C)]; therefore, all analyses were carried out on conformations sampled during the last 40 ns of the simulations.

The convergence of the simulations in this time window is further supported by the cosine content along the first eigenvector in both SERCA1a and PfATP6 trajectories. Indeed, the value of the cosine content in the last 40 ns is always below 0.5, being 0.41 and 0.46 for the whole proteins and 0.31 and 0.20 for the CPA binding sites on SERCA1a and PfATP6, respectively

### Binding energy calculation and structural/chemical features of the binding

The CPA binding site is located between two pairs of trans-membrane helices, M1-M2 and M3-M4 [Fig. 1(B)]. The residues in these helices stabilize the binding of the drug by establishing several interactions. The role of a divalent metal ion (Mg^2+^ or Mn^2+^) is also crucial for the stabilization of CPA into the binding site.^5^ In the CPA-SERCA1a co-crystal structure, residues _m_Gln56 and _m_Asp59 are mainly involved, together with three water molecules, in coordinating the manganese.

The absolute mean binding energy values of the CPA-SERCA1a and CPA-PfATP6 systems are -11.76 Kcal M^−1^ and −9.39 Kcal M^−1^, respectively. Importantly, these values, which do not differ significantly to each other (the standard deviation is 2.9 Kcal M^−1^ in both cases), are consistent with the observed experimental IC_50_ in the μM range.^4^,^13^–^15^

**Figure 3:**
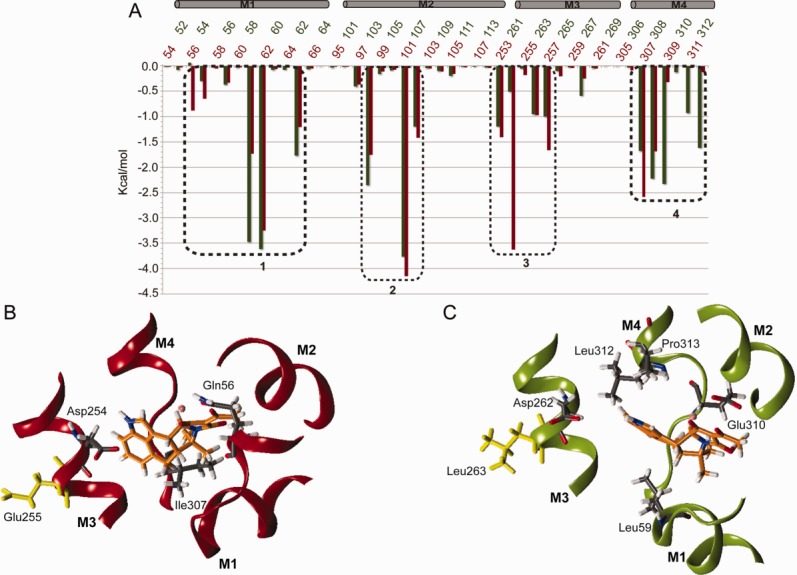
(A) Per-residue binding energy contribution calculated for the residues of the binding pocket in SERCA1a (red line) and PfATP6 (green line). The boxes on the top of the graph highlight the four helices forming the binding pocket. Dashed boxes highlight the four groups of amino acids that gave the highest energy contribution to CPA binding. Representative snapshot extracted after 145 ns of simulation time from the SERCA1a-CPA (B) and the PfATP6-CPA (C) trajectories showing the orientation of CPA in the binding pocket. [Color figure can be viewed in the online issue, which is available at http://wileyonlinelibrary.com.]

To understand whether there is any local difference between the CPA binding mode in PfATP6 and SERCA1a, we computed the per-residue binding energy contribution using the MM-GBSA method [Fig. 3(A)]. This approach is one of the most efficient post processing methods for free-energy and relative binding affinities estimation.^36^ As displayed in Figure 3(A), the profile of the per-residue energy contribution is overall similar in PfATP6 and SERCA1a except for some specific residues. More in detail, in both pumps the main energy contributions to the CPA binding come from four separate groups of amino acids, each group belonging to a different helix [dashed boxes in Fig. 3(A)]. The contribution of the residues belonging to helix M2 is conserved in the two proteins, whilst the relevant differences are found in helices M1, M3, and M4 [Fig. 3(A)]. More specifically, in SERCA1a, residues _m_Gln56 (helix M1), _m_Asp254 (helix M3) and _m_Ile307 (helix M4) contribute more to the CPA binding energy than the corresponding residues in PfATP6, whereas in PfATP6, residues _p_Leu59 (helix M1), and _p_Glu310, _p_Leu312, and _p_Pro313 (helix M4) play a more prominent role than their counterparts in SERCA1a [Fig. 3(A) and Table I].

**Table I:** Per Residue Energy Contribution to the CPA Binding in Kcal mol^−1^

	SERCA1a		PfATP6	
Helix	Residue	Energy contribution	Residue	Energy contribution
M1	_m_Gln56	−0.8	_p_Gln54	0.0
M2	_m_Asp254	−3.2	_p_Asp262	−0.2
M4	_m_Ile307	−2.6	_p_Ile308	−1.6
M1	_m_Leu61	−1.7	_p_Leu59	−3.5
M4	_m_Pro309	0.0	_p_Glu310	−2.4
M4	_m_Leu311	0.0	_p_Leu312	−0.9
M4	_m_Pro312	0.0	_p_Pro312	−1.6

**Figure 4:**
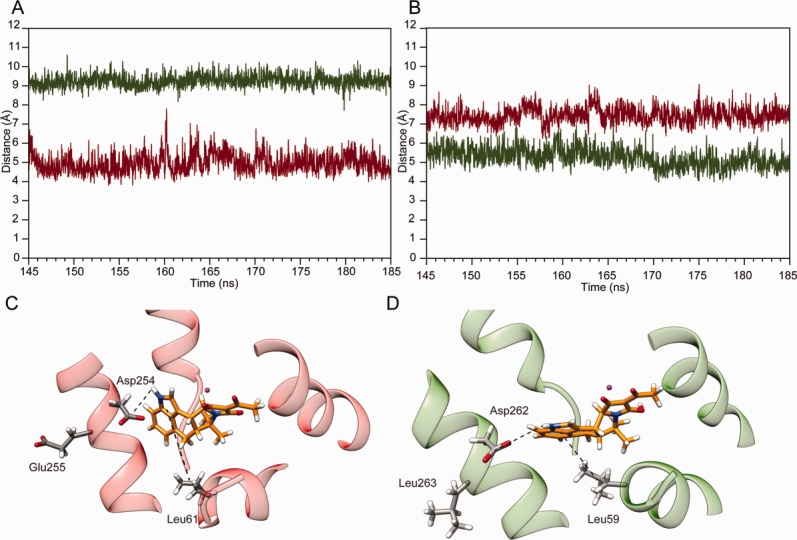
(A) Simulation time evolution of the distance between the hydrogen bound to the nitrogen atom N2 of CPA and the centre of mass of the carboxylic group of _m_Asp254 (red line) and _p_Asp262 (green line) and (B) of the centre of masses of the D and E rings of CPA with _m_Leu61 (red line) and _p_Leu59 (green line). Representative snapshot of CPA into the binding pocket of SERCA1a (C) and PfATP6 (D). The hydrogen bonds are shown as dashed lines. [Color figure can be viewed in the online issue, which is available at http://wileyonlinelibrary.com.]

After 145 ns of simulation time, the orientation of the residues' side-chain in the binding site had not changed, whilst the CPA binding mode looked different in the two proteins [Fig. 3(B,C)]. When bound to SERCA1a, CPA acquires a non-planar conformation where the D and E rings lay parallel to the M3 helix axis [Fig. 3(B)]. In contrast, in PfATP6, CPA has a planar structure where the D and E rings are roughly perpendicular to the M3 helix axis [Fig. 3(C)]. As shown in Figure 3(B,C), these differences are due to different CPA interaction patterns in the two proteins [Fig. 3(B,C)]. Indeed, the MM-GBSA calculations, together with a geometric analysis, allowed us to describe the peculiarities of the CPA binding in SERCA1a and PfATP6. We observed that the single glutamic acid to leucine non-conservative substitution (i.e., _m_E255 in SERCA1a and _p_Leu263 in PfATP6) induces a change in the CPA orientation in the binding pocket [Fig. 3(B,C)]. In particular, the distance between the center of mass of the carboxylic group of the aspartate residue _m_Asp254 or _p_Asp262 and the hydrogen bound to the nitrogen atom N2 of CPA, averaged over the last 40 ns of simulation time, is ∼4.5 Å in SERCA1a [Fig. 4(A), red line and 4(C)], and reaches ∼9.0 Å in PfATP6 [Fig. 4(A), green line and Fig. 4(D)]. On the other hand, the average distance between the center of mass of the methyl group of the leucine residue _m_Leu61 or _p_Leu69 and the D-E rings of CPA is ∼7.5 Å in SERCA1a and ∼5.2 Å in PfATP6 [Fig. 4(B) red vs. green line and Fig. 4(C,D)]. These results indicate that during the simulation the aspartic acid in the SERCA1 binding pocket (_m_Asp254) is on average closer to CPA than the corresponding residue (_p_Asp262) in PfATP6. This can be easily explained by the nature of the residue occupying a position spatially close to _m_Asp254 or _p_Asp262 which is a glutamic acid in SERCA1 (_m_Glu255) and a leucine in PfATP6 (_p_Leu263). In fact, the side chains of the two adjacent SERCA1a negatively charged residues, _m_Asp254 and _m_Glu255, are likely to repulse each other. This is supported by the fact that, during the simulation, the average distance between the centers of mass of the carboxylic groups of _m_Glu255 and _m_Asp254 is rather large (∼8.5 Å) (Supporting Information Fig. S4, red line). This repulsive effect can facilitate the electrostatic interaction of the carboxylic group of _m_Asp254 with the N2 nitrogen atom of CPA [Figs. 1(C) and 4(C)]. Noticeably, such electrostatic interaction accounts for a high contribution to the binding energy [∼−3.2 Kcal mol^−1^, Fig. 3(A) and Table I] and may represent the main cause for the different orientation of the D and E rings of CPA in the two binding sites [Fig. 4(C)].

In PfATP6, where the glutamic acid in SERCA1a is replaced by a leucine, the average distance between the centers of mass of the carbon δ1 and δ2 of _p_Leu263 and the carboxyl group of _p_Asp262 is shorter (∼2.5 Å) (Supporting Information Fig. S4, green line), suggesting that the two residues do not repulse each other. In absence of a repulsive effect, _p_Asp262 cannot establish interactions with the CPA, which, as a result, tends to assume a planar conformation and be closer to _p_Leu59 and, in general, to residues belonging to helix M4. Consistently, the CPA binding mode in PfATP6 is manly stabilized by hydrophobic interactions between _p_Leu59 and the indole moiety of the compound (D and E rings) [Fig. 4(D)] and by contacts with the three residues _p_Glu310, _p_Leu312 and _p_Pro313 of helix M4 which are exposed toward the Ca^2+^ channel.

## DISCUSSION

The regulation of Ca^2+^ homeostasis is essential for life in both prokaryotes and eukaryotes. Cyclopiazonic acid (CPA) is a potent inhibitor of the sarcoplasmic reticulum Ca^2+^-ATPase (SERCA), which is a protein responsible for the active transport of Ca^2+^ ions across the membrane by a mechanism coupled to ATP hydrolysis. The molecule binds into the Ca^2+^ channel and blocks the ion transport.

Using a computational approach, it has been possible to describe and interpret the binding mode of CPA in SERCA1a and PfATP6, the mammalian and *P. falciparum* Ca^2+^-ATPases, respectively. Despite the composition of the CPA binding site is highly conserved in the two proteins, a single non-conservative substitution, corresponding to _m_Glu255 in SERCA1a and to _p_Leu263 in PfATP6, occurs in a position adjacent to the binding site (Supporting Information Fig. S1, Figs. 3(B,C), 4(C,D)]. The effects of such substitution have been investigated in relation to the sensitivity of Ca^2+^-ATPases to artemisinin,^9^ whereas no studies exist that discuss how this amino acid change in SERCA1a position 255 affects the binding of CPA. Interestingly, Pulcini *et al*.^9^ recently found that the PfATP6 _p_Leu263Glu_p_ mutant (corresponding to position 255 in SERCA1a) shows an increased sensitivity to CPA.

To rationalize these observations and investigate the molecular mechanisms of the CPA binding to wild type SERCA1a and PfATP6, we modeled the PfATP6 structure using the crystal structure of SERCA1a as template and ran molecular dynamics simulations on both proteins immersed in a lipid bilayer [Fig. 1(A)].

The structural and energetic analyses of the simulation results showed that _m_Glu255 in SERCA1a and _p_Leu263 in PfATP6 indirectly affect the binding of CPA to the Ca^2+^-ATPases. Indeed, despite the fact that the binding of CPA is very stable in both systems, in the mammalian protein the electrostatic repulsion between the side chains of residues _m_Asp254 and _m_Glu255 (Supporting Information Fig. S4) leads to a favorable interaction between _m_Asp254 and CPA [Fig. 3(A,B) and Table I] which is not observed in PfATP6, where _m_Glu255 is replaced by a leucine and the electrostatic repulsion is not present (Supporting Information Fig. S4). The different arrangement of the interactions observed in the SERCAa1 and PfATP6 complexes is reflected by the different conformations adopted by CPA in the two binding pockets, being non-planar in SERCAa1 and planar in PfATP6.

In a recent work,^37^ the CPA-SERCA interactions essential for the compound inhibitory action, were identified by comparing the CPA binding site in the *Listeria monocytogenes* Ca^2+^-ATPase (LMCA1), a bacterial SERCA which is not inhibited by CPA, and in the mammal and plasmodial counterparts, that is, SERCA1a and PfATP6, respectively. The authors report that, despite the high overall sequence identity (38%), LMCA1 is inhibited by CPA at much higher concentrations than what is observed for SERCA1a. Therefore, they built a multiple sequence alignment including both CPA-sensitive and -insensitive P-type ATPases to analyse the conservation of amino acids located within 5 Å from CPA in the CPA-SERCA1a complex. This procedure made it possible to identify four positions varying in CPA-sensitive and -insensitive P-type ATPases: that is, _m_Gln56, _m_Leu61 (corresponding to _p_Leu59), _m_Gly257 and _m_Pro312. Our results not only are consistent with these findings, but also emphasize the importance of _p_Leu59 in stabilizing the CPA indole moiety for PfATP6 inhibition.

Furthermore, Woeste *et al*.^38^ showed that some bisphenols (BPs), which are composed of two hydroxylated phenyl groups typically linked through a methylene bridge, inhibit rabbit SERCA at low micromolar concentrations. A docking study from these authors, suggested that the BP most non-polar ring partly occupies the CPA binding pocket, in close proximity to residues _m_Leu61, _m_Val62, _m_Leu65, _m_Leu253, _m_Pro308, and _m_Pro312 (corresponding to PfATP6 _p_Leu59, _p_Val60, _p_Leu63, _p_Ile261, _p_Pro309, and _p_Pro313, respectively), whereas the other ring protrudes in a different pocket establishing a single hydrogen bond with _m_Asp254 (corresponding to _p_Asp256 in PfATP6). This indicates that, also in the case of BP, the network of hydrophobic contacts with residues belonging to helices M3 and M4—including _m_Leu61—is one of the major driving force in the inhibitor binding.

**Figure 5:**
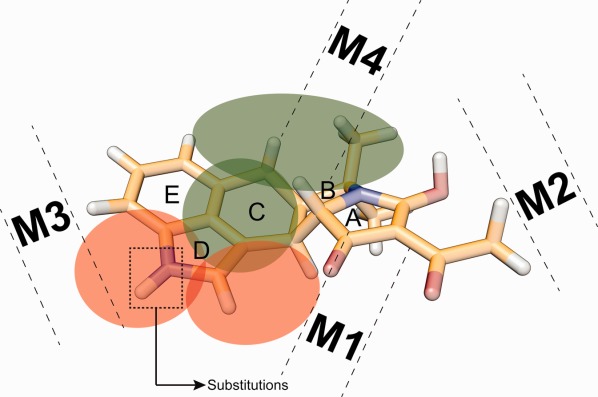
Schematic representation of the CPA regions involved in favorable interactions in the two pumps. The red shaded circle highlights CPA regions establishing more favorable interactions in SERCA1a than in PfATP6 whereas the green shaded circle indicate the drug regions establishing more favorable interactions in PfATP6 than in SERCA1a. The dashed rectangle highlights a potentially optimal site for the introduction of a substituent capable of disrupting the interaction with _m_Asp254. [Color figure can be viewed in the online issue, which is available at http://wileyonlinelibrary.com.]

The main differences between SERCA1a and PfATP6 in the CPA binding are schematically reported in Figure 5, where the drug regions establishing more favorable interactions in SERCA1a (PfATP6) than in PfATP6 (SERCA1a) are in red- (green-) shaded circles.

To increase the specificity of CPA toward PfATP6, substituents destabilizing favorable CPA-SERCA1a interactions could be introduced. For example, the introduction of a large and negatively charged group at the nitrogen atom position in the CPA D ring (dashed box in Fig. 5) would likely prevent a stable interaction of the drug with _m_Asp254 through steric hindrance, on one hand, and an increased repulsive force, on the other.

## CONCLUSION

CPA, which is produced by some molds, can contaminate animal feeds and food sources, but is also a potent inhibitor of PfATP6. However, CPA is not selective against the Ca^2+^ pump of *P. falciparum*, indeed it also binds and inhibits the mammalian SERCA. For this reason, the design and synthesis of derivatives that can act with the same molecular mechanism without blocking the mammalian Ca^2+^ pump are desirable. Our computational approach allowed a thorough characterization of the CPA binding to wild type SERCA1a and PfATP6 at the atomic level and highlighted specific characteristics of the different modes of CPA binding to the mammalian and plasmodial Ca^2+^ pumps. The conclusions derived from this work can help direct the design and synthesis of selective inhibitors of PfATP6 with a reduced activity against mammalian SERCA.
