# Supplementary material for: Characterization of the differences in the cyclopiazonic acid binding mode to mammalian and P. Falciparum Ca2+ pumps: A computational study
Source: Proteins. 2015 Jan 10;83(3):564–74. doi: 10.1002/prot.24734 (PMC4342768; doi:10.1002/prot.24734)
Supplement: Supplementary file 1 — Supporting Information [file prot0083-0564-sd1.pdf]

Characterization of the differences in the cyclopiazonic acid binding mode to mammalian and *P. falciparum* Ca^{2+} pumps: a computational study

Daniele Di Marino[1], Ilda D'Annessa[2], Andrea Coletta[2], Allegra Via[1*], Anna Tramontano[1,3]

Supporting Information

A

```
                     M1                                      M2
Homo sapiens         LWELVLEQFEDLLVRILLLAALVSFVLAWFEEGEET--TTAFVEPLVIMLILVANAIVGVWQE 108
Oryctolagus cuniculu LWELVIEQFEDLLVRILLLAACISFVLAWFEEGEET--ITAFVEPFVILLILIANAIVGVWQE 108
Makaira nigrica      IWDLIVEQFEDLLVRILLLAACISFVLAWFEEGEET--ITAFVEPFVILLILIANAIVGVWQE 108
Pinctada fucata      LWELILEQFDDLLVKILLLAAIISFVLAWFEESEEQ--VTAFVEPFVILTILICNAVVGVWQE 108
Plasmodio falciparum IFELILNQFDDLLVKIILLLAAFISFVLTLLDMKXKKIEICDFIEPLVIVLILILNAAVGVWQ 108
Procambarus clarkii  LLQLILEQFYDLLVKILLLAAIISFVLACFEEGEET--VTAFVEPFVILLILIANAIVGVWQE 108
                     : :*:::** ****:****** :****: ::   :      *:**:**: **: ** ******
                     M3                                      M4
Homo sapiens         TPLQRKLDEFGRQLSHAISVICVAVWVINIGHFADPAHGGSWLRGAVYYFKIAVALAVAAIPEGLPAVITT 317
Oryctolagus cuniculu TPLQQKLDEFGEQLSKVISLICVAVWLINIGHFNDPVHGGSWIRGAIYYFKIAVALAVAAIPEGLPAVITT 317
Makaira nigricans    TPLQAKLDEFGEQLSKVISLICVAVWAINIGHFNDPVHGGSWIRGAVYYFKIAVALAVAAIPEGLPAVITT 317
Pinctada fucata      TPLQQKLDEFGQQLSKVITVICVAVWAINIGHFNDPAHGGSWIKGAIYYFKIAVALAVAAIPEGLPAVITT 317
Plasmodio falciparum TPLQIKIDLFGQQLSKIIFVICVTVWIINFKHFSDPIX-GSFLYGCLYYFKISVALAVAAIPEGLPAVITT 317
Procambarus clarkii  TPLQQKLDEFGEQLSKVISIICVAVWAINIGHFNDPAHGGSWIKGAIYYFKIAVALAVAAIPEGLPAVITT 317
                     **** *:* **.***: * :***:** **:  * **   **:: *.:*****:******************
                     M5                                M6
Homo sapiens         YSNMKQFIRYLISSNVGEVVCIFLTAILGLPEALIPVQLLWVNLVTDGLPATALGF 782
Oryctolagus cuniculu YNNMKQFIRYLISSNVGEVVCIFLTAALGLPEALIPVQLLWVNLVTDGLPATALGF 782
Makaira nigricans    YNNMKQFIRYLISSNVGEVVCIFLTAALGLPEALIPVQLLWVNLVTDGLPATALGF 779
Pinctada fucata      YNNMKQFIRYLISSNIGEVVCIFLTAALGIPEALIPVQLLWVNLVTDGLPATALGF 782
Plasmodio falciparum YNNMKAFIRYLISSNIGEVASIFITALLGIPDSLAPVQLLWVNLVTDGLPATALGF 992
Procambarus clarkii  YNNMKQFIRYLISSNVGEVVSIFLTAALGLPEALIPVQLLWVNLVTDGLPATALGF 782
                     *.*** *********:***..**:** **:*::* ********************
                     M7                                M8
Homo_sapiens         LISGWLFFRYLAIGVYVGLATVAAATWWFV   857   PTTMALSVLVTIEMCNALNSVSENQS   920
Oryctolagus cuniculu LISGWLFFRYMAIGGYVGAATVGAAAWWFM   857   PMTMALSVLVTIEMCNALNSLSENQS   920
Makaira nigricans    LISGWLFFRYMAIGGYVGAATVGGAAWWFL   857   PMTMALSVLVTIEMCNALNSLSENQS   917
Pinctada fucata      LITGWLFFRYMAIGIYVGCATVGAAAWWFM   857   PMTMALSVLVTIEMLNALNSLSENQS   920
Plasmodio falciparum LINGLTLLRYIIIGTYVGIATVSIFVYWFL  1067   PMTMALSVLVTIEMCNALNSLSENQS  1143
Procambarus clarkii  LISGWLFFRYMAIGGYVGAATVFAASWWFM   857   PMTMALSVLVTIEMLNALNSLSENQS   920
                     **.*  ::**: ** *** ***    :**:         ************* *****:******
                     M9                                M10
Homo sapiens         NPWLLVAVAMSMALHFLILLVPPLPLIFQVTPLSGRQWVVVLQISLPVILLDEALKYL   987
Oryctolagus cuniculu NIWLLGSICLSMSLHFLILYVDPLPMIFKLKALDLTQWLMVLKISLPVIGLDEILKFI   987
Makaira nigricans    NLWLMAAMTLSMSLHFMIIYVDPLPMIFKLTHLTFDQWLMVFKLSFPVILIDEVLKFF   984
Pinctada fucata      NKWLLGAIALSMSLHFFILYVDVMSTIFQITPLNVAEWIAVLKISIPVIILDETLKFI   987
Plasmodio falciparum NIWLLGSICLSMSLXFLILYVDPLPMIFKLKALDLTQWLMVLKISLPVIGLDEILKFY  1211
Procambarus clarkii  NFWLLAAMALSMTLHFIILYIDILSTVFQVMPLSVAQWVAVLKISFPVLLLDETLKFI   987
                     * **: :: :**:* *:*: :  :. :*::  *   :*: *:::*:**: :** **::
```

B

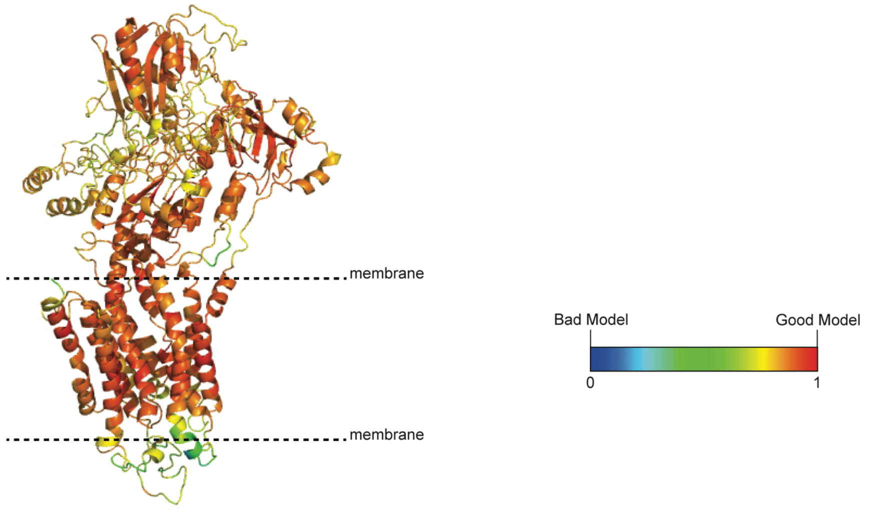

Figure S1. (**A**) Multi-sequence alignment of the transmembrane (TM) region comprising sequences coming from different species. The boxes at the top of the alignment indicate the TM helices. The rectangular box highlights the glutamic acid conserved in the sequences from all species but *P. falciparum* and indirectly involved in the binding of CPA. (**B**) Three-dimensional arrangement of the TM bundle of helices in the X-ray structure of rabbit SERCA (PDB: 3FGO). Ribbon representation of the PfATP6 model built using the X-ray structure of rabbit SERCA1a (PDB: 3FGO) as template. The structure is colored according to the color scale of QMEANBrane, the quality estimation method for membrane proteins used to evaluate the quality of the PfATP6 model.

```
P. falciparum    1 MEEVIKNAHTYDVEDVLKFLDVNKDNGLKNEELDDRRLKYGLNELEVEKK   50
                   ||.    ||:...|:.|.:..|::..||..::.....|||.|||..|:.
Rabbit           1 MEA----AHSKSTEECLAYFGVSETTGLTPDQVKRHLEKYGHNELPAEEG   46

P. falciparum   51 KSIFELILNQFDDLLVKILLLAAFISFVLTLLDMKHKKIEICDFIEPLVI  100
                   ||::||::.||:||||:||||||.|||||...:...:.|..  |:||.||
Rabbit          47 KSLWELVIEQFEDLLVRILLLAACISFVLAWFEEGEETITA--FVEPFVI   94

P. falciparum  101 VLILILNAAVGVWQECNAEKSLEALKELQPTKAKVLRDGKWEI--IDSKY  148
                   :||||.||.||||||.|||.::|||||.:|...||.|..:..:  |.::.
Rabbit          95 LLILIANAIVGVWQERNAENAIEALKEYEPEMGKVYRADRKSVQRIKARD  144

P. falciparum  149 LYVGDIIELSVGNKTPADARIIKIYSTSLKVEQSMLTGESCSVDKYAEKM  198
                   :..|||:|::||:|.|||.||:.|.||:|:|:||:|||||.||.|:.|.:
Rabbit         145 IVPGDIVEVAVGDKVPADIRILSIKSTTLRVDQSILTGESVSVIKHTEPV  194

P. falciparum  199 EDSYKNCEIQLKKNILFSSTAIVCGRCIAVVINIGMKTEIGHIQHAVIES  248
                   .|  .....|.|||:|||.|.|..|:.:.:|...|:.||||.|:..:  :
Rabbit         195 PD--PRAVNQDKKNMLFSGTNIAAGKALGIVATTGVSTEIGKIRDQM--A  240

P. falciparum  249 NSEDTQTPLQIKIDLFGQQLSKIIFVICVTVWIINFKHFSDPIHG-SFLY  297
                   .:|..:||||.|:|.||:||||:|.:|||.||:||..||:||:|| |::.
Rabbit         241 ATEQDKTPLQQKLDEFGEQLSKVISLICVAVWLINIGHFNDPVHGGSWIR  290

P. falciparum  298 GCLYYFKISVALAVAAIPEGLPAVITTCLALGTRRMVKKNAIVRKLQSVE  347
                   |.:|||||:|||||||||||||||||||||||||||.|||||||.|.|||
Rabbit         291 GAIYYFKIAVALAVAAIPEGLPAVITTCLALGTRRMAKKNAIVRSLPSVE  340

P. falciparum  348 TLGCTTVICSDKTGTLTTNQMTTTVFHLFRESD----SLTEYQLCQKGDT  397
                   |||||:|||||||||||||||:.....:..:.|    ||.|:.:  .|.|
Rabbit         341 TLGCTSVICSDKTGTLTTNQMSVCKMFIIDKVDGDFCSLNEFSI--TGST  380

P. falciparum  398 YYFYESSNLTNDIYAGESSFFNKLKDEGNVEALTDDGEEGSIDEADPYSD  447
                   |.  |....||      .|. |: | : ..
Rabbit         381 Y-APEGEVLKNDKPI-RSGQFDGLVELAT---------------------  395

P. falciparum  448 YFSSDSKKMKNDLNNNNNNNNNNSSRSGAKRNIPLKEMKSNENTIISRGSK  497

Rabbit         396 --------------------------------------------------  415

P. falciparum  498 ILEDKINKYCYSEYDYNFYMCLVNCNEANIFCNDNSQIVKKFGDSTELAL  547
                                      :|.: ||::::..|:...:.:|.|::||.||
Rabbit         416 -------------------ICAL-CNDSSLDFNETKGVYEKVGEATETAL  445

P. falciparum  548 LHFVHNFDILPTFSKNNKMPAEYEKNTTPVQSSNKKDKSPRGINKFFSSK  597
                   ||.|.::::: |  :|
Rabbit         446 TTLVEKMNVFNTEVRNLSKV------------------------------  453

P. falciparum  598 NDNSHITSTLNENDKNLKNANHSNYTTAQATTNGYEAIGENTFEHGTSFE  647

Rabbit         454 --------------------------------------------------  469

P. falciparum  648 NCFHSKLGNKINTTSTHNNNNNNNNNSNSVPSECISSWRNECKQIKIIEF  697
                                                   .|.|..|...|:...:||
Rabbit         470 ----------------------------ERANACNSVIRQLMKKEFTLEF  491

P. falciparum  698 TRERKLMSVIVENKKKEII-----LYCKGAPENIIKNCKYYLTKNDIRPL  742
                   :|:||.|||.....|....     ::.|||||.:|..|.|........|:
Rabbit         492 SRDRKSMSVYCSPAKSSRAAVGNKMFVKGAPEGVIDRCNYVRVGTTRVPM  541

P. falciparum  743 NETLKNEIHNKIQNMG--KRALRTLSFAYKKLSSK--DLNIKNTDDYYKL  788
                   ...:|.:|.:.|:..|  :..||.|:.|.:....|  ::.:.::..:.:.
Rabbit         542 TGPVKEKILSVIKEWGTGRDTLRCLALATRDTPPKREEMVLDDSSRFMEY  591

P. falciparum  789 EQDLIYLGGLGIIDPPRKYVGRAIRLCHMAGIRVFMITGDNINTARAIAK  838
                   |.||.::|.:|::|||||.|..:|:||..|||||.||||||..||.||.:
Rabbit         592 ETDLTFVGVVGMLDPPRKEVMGSIQLCRDAGIRVIMITGDNKGTAIAICR  641

P. falciparum  839 EINILNKNEGDDEKDNYTNNKNTQICCYNGREFEDFSLEKQKHILKNTPR  888
                   .|.|..:||...::            .|.||||:|..|.:|:...:..
Rabbit         642 RIGIFGENEEVADR------------AYTGREFDDLPLAEQREACRRA--  677

P. falciparum  889 IVFCRTEPKHKKQIVKVLKDLGETVAMTGDGVNDAPALKSADIGIAMGIN  938
                   ..|.|.||.||.:||:.|:...|..||||||||||||||.|:|||||| :
Rabbit         678 CCFARVEPSHKSKIVEYLQSYDEITAMTGDGVNDAPALKKAEIGIAMG-S  726

P. falciparum  939 GTEVAKEASDIVLADDNFNTIVEAIKEGRCIYNNMKAFIRYLISSNIGEV  988
                   ||.|||.||::|||||||:|||.|::|||.||||||.|||||||||:|||
Rabbit         727 GTAVAKTASEMVLADDNFSTIVAAVEEGRAIYNNMKQFIRYLISSNVGEV  776

P. falciparum  989 ASIFITALLGIPDSLAPVQLLWVNLVTDGLPATALGFNPPEHDVMKCKPR 1038
                   ..||:||.||:|::|.|||||||||||||||||||||||||:.|:|...||
Rabbit         777 VCIFLTAALGLPEALIPVQLLWVNLVTDGLPATALGFNPPDLDIMDRPPR  826

P. falciparum 1039 HKNDNLINGLTLLRYIIIGTYVGIATVSIFVYWFLFYPDSDMHTLINFYQ 1088
                   ...:.||:|....||:.||.|||.|||....:||::..|...   :.::|
Rabbit         827 SPKEPLISGWLFFRYMAIGGYVGAATVGAAAWWFMYAEDGPG---VTYHQ  873

P. falciparum 1089 LSHYNQCKAWNNFRVNKVYDMSEDHCSYFSAGKIKASTLSLSVLVLIEMF 1138
                   |:|:.||..      :..:....| |..|.|.  :..|::|||||.|||.
Rabbit         874 LTHFMQCTE------DHPHFEGLD-CEIFEAP--EPMTMALSVLVTIEMC  914

P. falciparum 1139 NALNALSEYNSLFEIPPWRNMYLVLATIGSLLLHVLILYIPPLARIFGVV 1188
                   ||||:|||..||..:|||.|::|:.:...|:.||.||||:.||..||.:.
Rabbit         915 NALNSLSENQSLMRMPPWVNIWLLGSICLSMSLHFLILYVDPLPMIFKLK  964

P. falciparum 1189 PLSAYDWFLVFLWSFPVIILDEIIKFYAKRKLKEEQRTKKIKID 1228
                   .|....|.:|...|.|||.||||:||.|:..|::.:.::
Rabbit         965 ALDLTQWLMVLKISLPVIGLDEILKFIARNYLEDPEDERR---K 1001
```

Figure S2. Pairwise sequence alignment of the target (PfATP6) and template (rabbit SERCA1a) proteins.

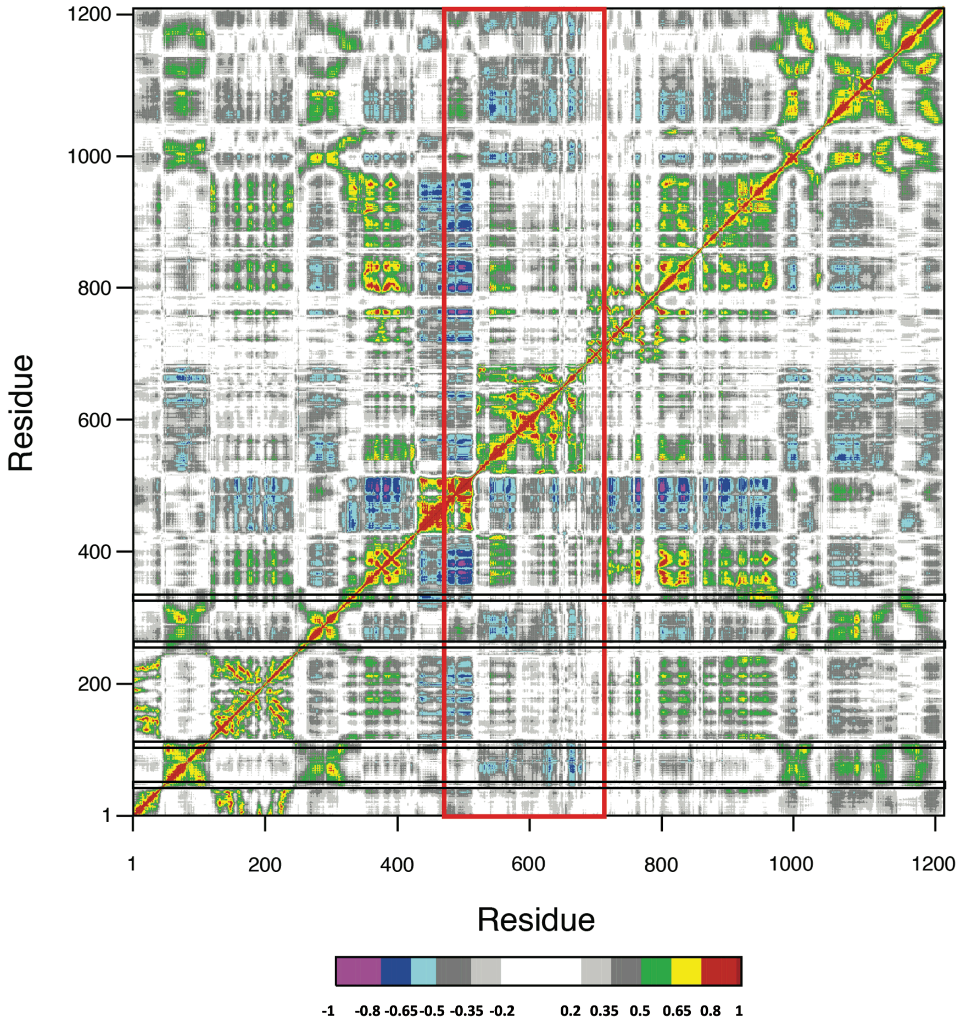

Figure S3. Cross correlation matrix calculated for the Cα atoms of PfATP6. The residues of the CPA binding site are highlighted by black rectangles and the residues belonging to the LCR regions by a red rectangle. White-colored matrix cells indicate uncorrelated residues.

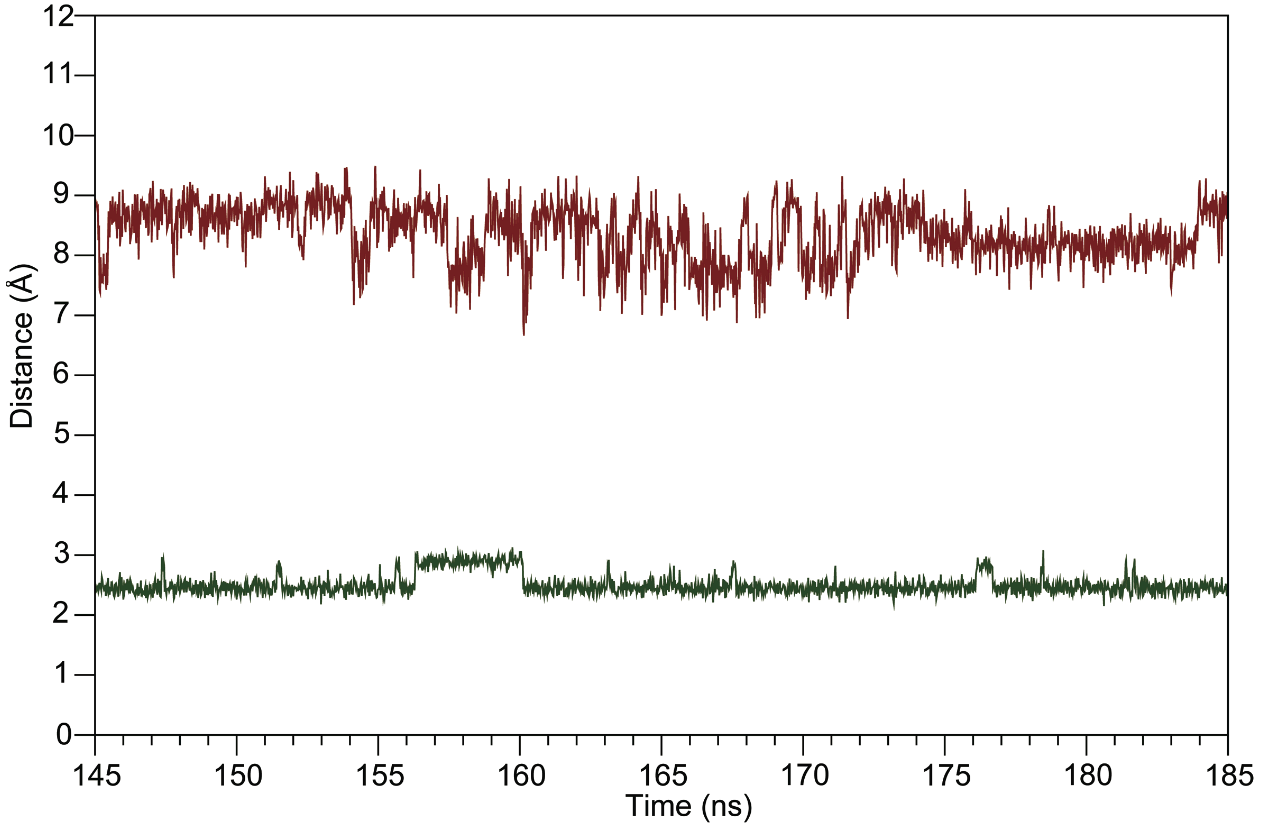

Figure S4. Evolution during the simulation time of the distance between the centers of masses of the carboxylic groups of $_m$Glu255 and $_m$Asp254 in SERCA1a (red line) and between the centers of masses of the carboxylic group of $_p$Asp262 and the δ1 and δ2 carbons of $_p$Leu263 of PfATP6 (green line).
